# One-time double-layer placement of controlled-release urea enhances wheat yield, nitrogen use efficiency and mitigates N_2_O emissions

**DOI:** 10.3389/fpls.2025.1634174

**Published:** 2025-08-22

**Authors:** Muhammad Akhtar, Wu Liuge, Chen Jian, Su Yuxiao, Zheng Yuntan, Lu Yulun, Zheng Shanchao, Deng Aixing, Song Zhenwei, Zheng Chengyan, Zhang Weijian

**Affiliations:** ^1^ Institute of Crop Sciences, Chinese Academy of Agricultural Sciences, Key Laboratory of Crop Physiology and Ecology, Ministry of Agriculture and Rural Affairs of China, Beijing, China; ^2^ Dongping County Agricultural Bureau, Tai'an, China

**Keywords:** layered fertilization, controlled-release urea, nitrogen use efficiency, wheat yield, N_2_O emissions

## Abstract

Simultaneously enhancing the crop yield and reducing nitrous oxide (N_2_O) emissions presents a critical challenge in sustainable agriculture. The application of nitrogen (N) fertilizer is a key strategy to enhance crop yield. However, conventional N application practices often lead to excessive soil N accumulation, insufficient crop N uptake and elevated greenhouse gas (GHG) emissions. To address these issues, this study evaluated the effectiveness of one-time double-layer fertilization of controlled-release urea (CRU) in improving wheat yield, nitrogen use efficiency (NUE) and mitigating N_2_O emissions compared to single-layer fertilization. A two-year field experiment (2021-2023) was conducted with five treatments: zero N fertilizer (T0), one-time single-layer fertilization of urea at 8–10 cm soil depth (T1), one-time single-layer fertilization of CRU at 8–10 cm soil depth (T2), one-time double-layer fertilization of urea at 8–10 cm & 18–20 cm soil depth (T3), one-time double-layer fertilization of CRU at 8–10 cm & 18–20 cm soil depth (T4). The two-year average results indicated that one-time double-layer fertilization of CRU (T4) achieved the highest wheat yield (10.20 t ha^-1^) and NUE (19.13 kg kg^-1^), as well as the lowest N_2_O emissions (0.66 kg ha^-1^). Compared to single-layer CRU fertilization (T2), T4 increased wheat yield and NUE by 5.94% and 11.26%, respectively, while reducing N_2_O emissions by 22.50%. Furthermore, T4 optimized the soil microenvironment by lowering soil temperature and NO_3_^−^-N content at 0–10 cm depth, while enhancing soil moisture and NH_4_^+^-N availability at 10–20 cm, thereby promoting plant N uptake and utilization. These findings suggest that the one-time double-layer fertilization of CRU synchronizes N release with crop demand and regulates soil N dynamics, offering a promising strategy to boost wheat productivity and minimize environmental impacts.

## 1 Introduction

Agriculture contributes 25-30% of global greenhouse gas emissions, with 60% of anthropogenic nitrous oxide (N_2_O) emissions arising from agricultural soils, accounting for 21% of total global N_2_O emissions (Aliyu et al., 2019; Ding et al., 2007; Vermeulen et al., 2012). N_2_O has a warming potential of 298 times larger than CO_2_, with significant negative impacts on health and stratospheric ozone depletion (Friedl et al., 2021; Hoben et al., 2011). The major source of agricultural N_2_O emissions is synthetic N fertilizers, whose use increased by 37% between 2001 and 2011 (Gerber et al., 2016) and will rise by 50% between 2000 and 2050 (Wang et al., 2024). The wheat yield in Northern China has significantly enhanced by 50% between 2000 and 2021, covering 58% of China’s overall wheat production and contributing 30% N_2_O (Gao et al., 2011).

Nitrogen (N) fertilizer plays a vital role in promoting wheat growth. However, when applied excessively, it can result in N loss and elevate N_2_O emissions (Gaihre et al., 2015; Liu et al., 2024a), due to an increase in the concentrations of N in the soil (Takeda et al., 2021). Urea, a widely used fertilizer, poses a significant environmental threat due to its characteristics and can easily escape into the atmosphere and water bodies (Reay et al., 2012). Conventional broadcast application of N fertilizer results in about 30% loss of N as gas, with N recovery efficiencies ranging from 30-45% (Gaihre et al., 2015). A single application of normal urea is insufficient to fulfill the crop N demand, excessive amounts of application during critical growth stages lead to N surplus in the soil and elevate the emissions of N_2_O (Zhang et al., 2019). Splitting N application during the entire crop growth period improves nutrient absorption and grain yield (Ma et al., 2021b). However, this approach involved challenges for farmers due to time-consuming and labor expenses (Li et al., 2018). Controlled-release urea (CRU) offers a promising solution to these challenges (Ke et al., 2018; Li et al., 2018). Research indicates that CRU provides numerous advantages, including labor and time savings through a single basal application, enhanced N use efficiency (NUE), and synchronization of N release with plant absorption (Geng et al., 2016; Hu et al., 2023a; Ye et al., 2013). Furthermore, it helps to minimize N losses, contributing to more sustainable agricultural practices (Zhang et al., 2019).

Crop production is a complicated process that emits gaseous N losses at each stage (Liu et al., 2022a). Some previous studies have indicated that the way crops absorb nutrients during various growth phases is connected to the distribution of their roots and shows changes over time (Chen et al., 2022; Liu et al., 2022c). At the initial growth (seedling stages), a single deep placement of fertilizer did not meet the crop N requirement and the roots mostly obtain nutrients from the soil layer that is 5 cm deep (Liu et al., 2024b). However, as the crop progresses to the anthesis stage, it relies on nutrients supplied by the soil layer at 25 cm depth (Chen et al., 2022; Liu et al., 2022c). Prior research found that deep application of CRU can significantly decrease the gaseous N loss and enhance NUE and yield of different food crops, including wheat (Chen et al., 2023; Rychel et al., 2020; Wu et al., 2021; Zhang et al., 2022). However, other researchers have reported conflicting outcomes, greater placement depth increased the N loss and reduced yield (Ke et al., 2018; Ma et al., 2021b; Wang et al., 2023). However, previous research suggests that layered fertilization provides significant benefits in stimulating root development and N absorption by optimizing the placement of N fertilizers over various soil layers rather than employing a single deep fertilization technique (He et al., 2024; Liu et al., 2024a; Wu et al., 2023). Nevertheless, crop yield and N_2_O emissions responses to one-time layer placement of different N fertilizers are still unclear, indicating the need for additional research for sustainable winter wheat production and environmental protection in North China.

A research trial was conducted to examine the impact of different N sources (urea vs. CRU) and fertilization strategies (single-layer vs. double-layer) on wheat grain production, N use efficiency, and N_2_O emissions. The findings from this study may provide practical recommendations for improving N management practices to enhance wheat productivity while mitigating N_2_O emissions.

## 2 Materials and methods

### 2.1 Design of experiment

A field trial was conducted between 2021–2023 in Dongping County, Shandong Province, located at coordinates 35°89’N, 116°36’E. The Jimai 22 variety of winter wheat was sown on October 30, 2021, and harvested on June 17, 2022, during the first-year experiment. In the second-year experiment, sowing occurred on October 15, 2022, and harvesting was completed on June 15, 2023. Before starting the experiment, the physicochemical characteristics of the subsurface soil at a depth of 20 cm were analyzed using standard laboratory methods. The soil organic matter (SOM) and total N were measured using an elemental analyzer (vario PYRO, Elementar, Germany). Available N was determined by using the alkaline hydrolysis diffusion method, available P was extracted using hydrochloric acid and sodium bicarbonate, followed by the molybdenum-antimony colorimetric method, while available K was measured through flame photometry. The tested soil at the experimental site was classified as fluvo-aquic, with a clay loam texture, typical of the Yellow River alluvial plain. The baseline physicochemical characteristics of the soil were as follows: SOM of 17.60 g kg^−1^, total N of 1.18 g kg^−1^, available N of 104.10 mg kg^−1^, available P of 40.48 mg kg^−1^, available K of 108.68 mg kg^−1^.

**Figure 1:**
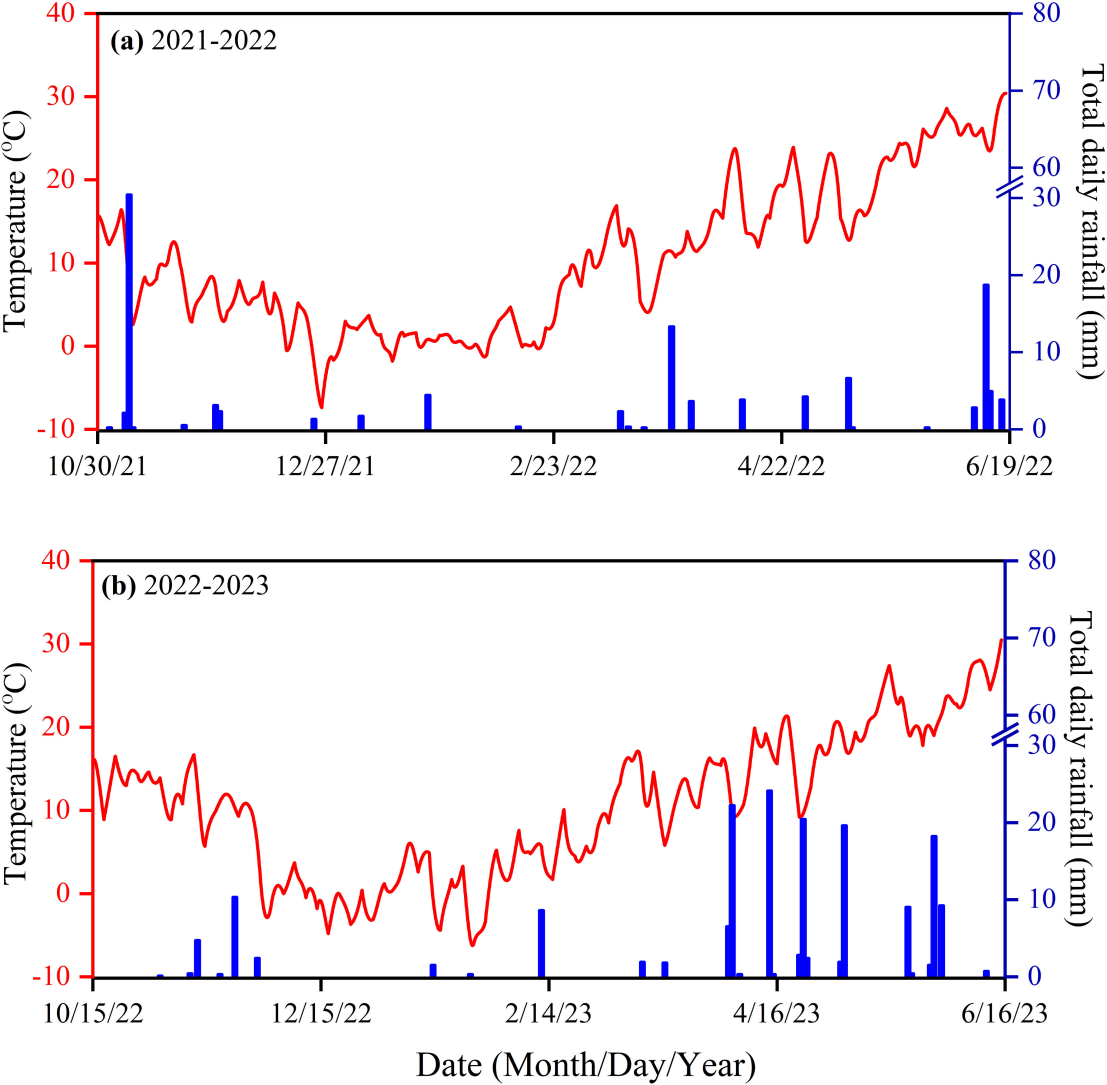
Daily average air temperature and rainfall during two wheat-growing periods, **(a)** 2021-2022, **(b)** 2022-2023.

**Figure 2:**
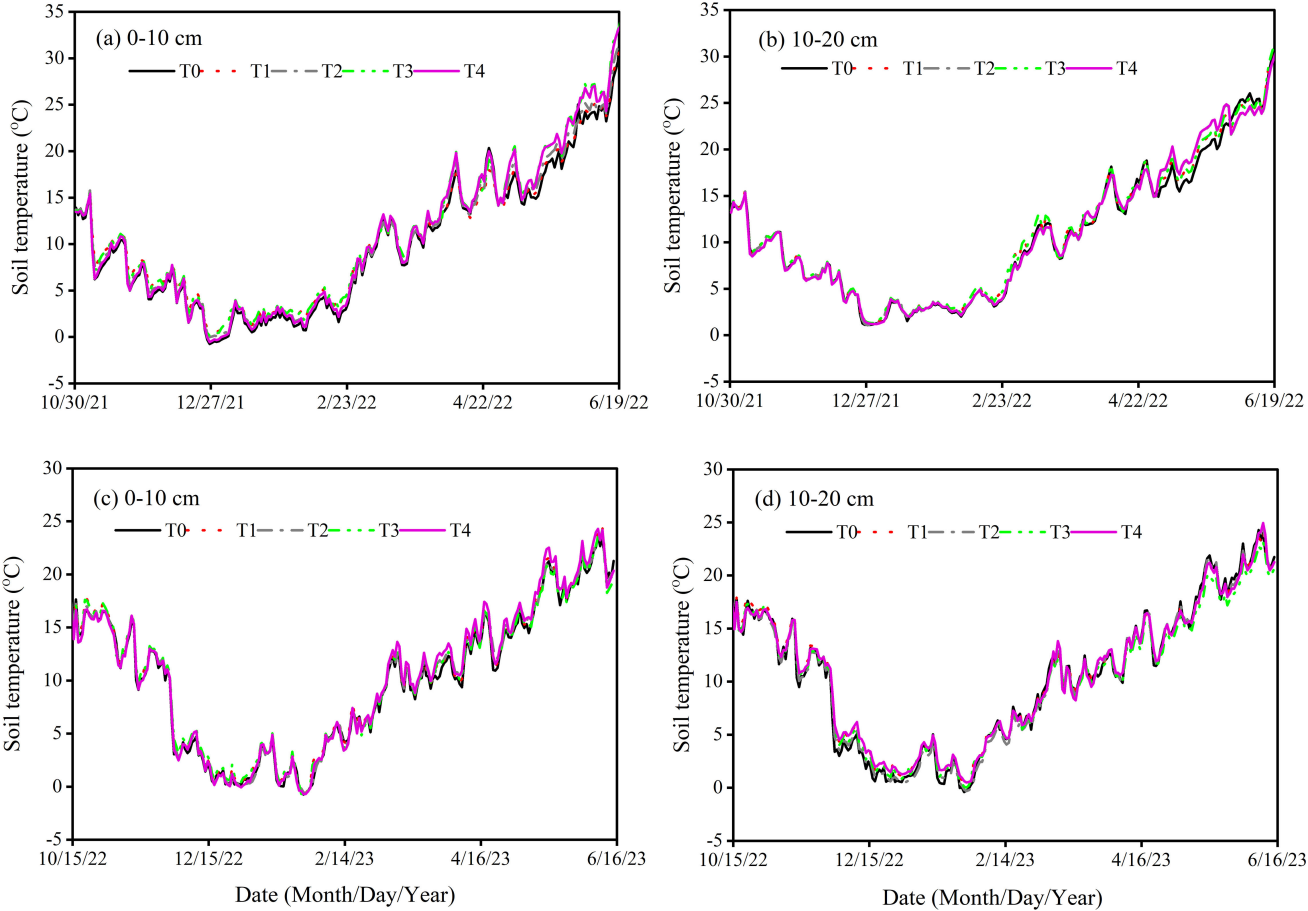
Soil temperature (°C) at 10 cm **(a, c)** and 20 cm **(b, d)** soil depth during winter wheat development under various treatments in 2021-2023.

**Figure 3:**
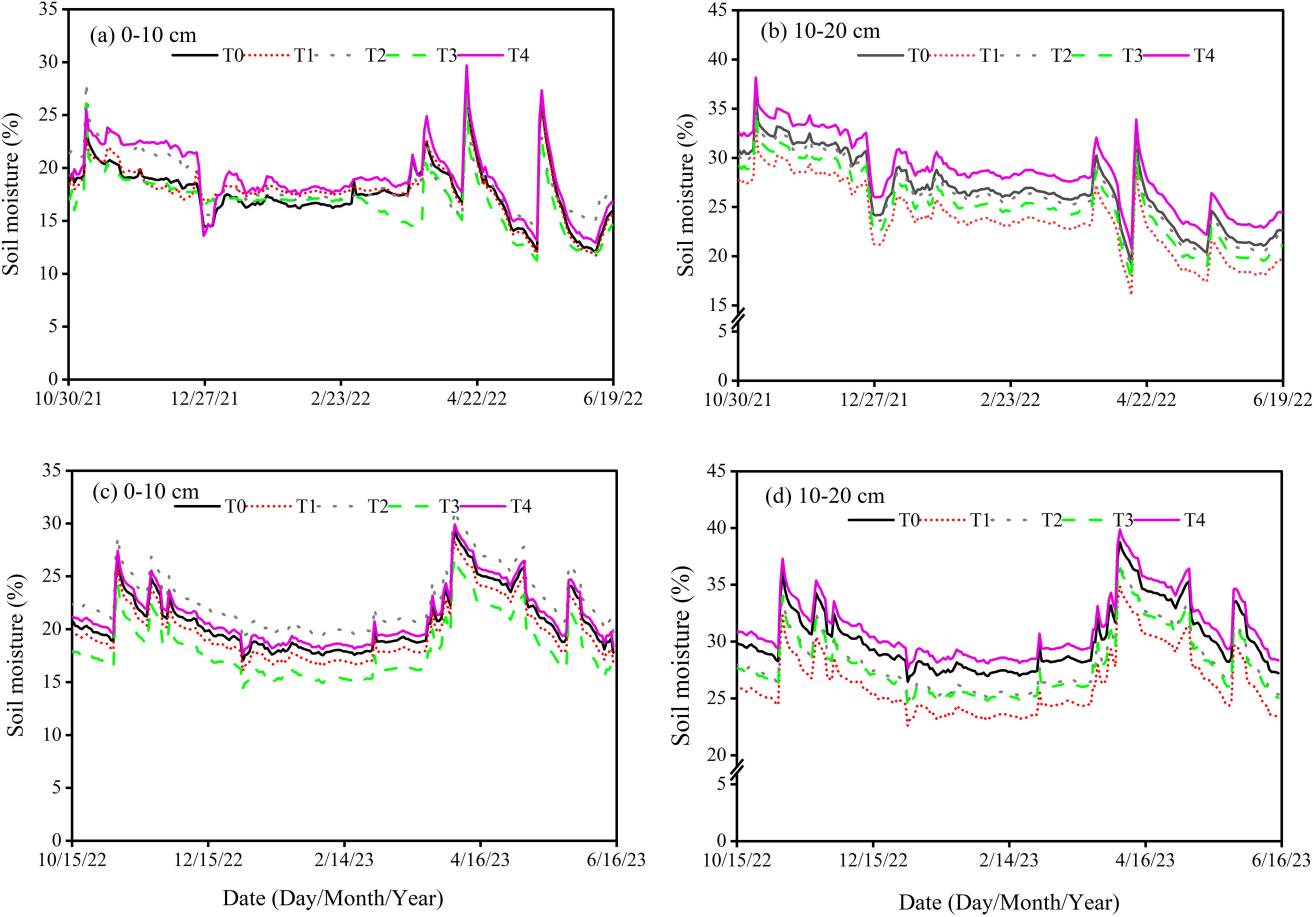
Soil moisture at 10 cm **(a, c)** and 20 cm **(b, d)** soil depth during winter wheat development under various treatments in 2021-2023.

To examine the impact of different N fertilizer types and layered fertilization, comprising five treatments were applied, as summarized in **Table 1**. The treatments were assigned randomly to three replicates, resulting in 15 plots. Each plot was 3 meters long and 2 meters wide, with a total size of 6 square meters. It was divided into 8 rows with 25 cm distance spacing in each row. Prior to planting, 2–3 rounds of rotary tillage were conducted to prepare a fine seedbed. The application rate of fertilizer was consistent across all treatments, except for the control, with N, P_2_O_5_, and K_2_O application rates at 240 kg ha^-1^, 120 kg ha^-1^, and 90 kg ha^-1^, respectively. The fertilizers utilized in this experiment included urea (46% N), CRU coated with polyurethane (44% N), calcium superphosphate (16% P_2_O_5_), and potassium chloride (16% K_2_O). Urea and CRU were applied as a basal application for both single-layer and double-layer treatments, while P_2_O_5_ and K_2_O were applied uniformly across all treatments, spread on the soil surface before tilling (Ali et al., 2025; Cui et al., 2022). Pesticides, fungicides, and herbicides were used uniformly to protect against diseases, insect pests and weeds. Sprinkler irrigation was applied during the jointing period. The weather data collected from the experimental site is illustrated in **Figure 1**. The soil temperature and moisture were measured at 10 and 20 cm soil depths by installing the automatic sensor of ZDR-U; ZEDA (**Figures 2**, **3**).

**Table 1:** Fertilizer application treatments for winter wheat experiment from 2021-2023.

Treatment	Fertilizer type	Application depth (cm)	N (kg ha^-1^)	P_2_O_5_ (kg ha^-1^)	K_2_O (kg ha^-1^)	N fertilizer (%)
T0	No N fertilizer	–	0	120	90	–
T1	Single-layer urea	8-10	240	120	90	Urea (46% N)
T2	Single-layer CRU	8-10	240	120	90	CRU (44% N)
T3	Double-layer urea	8-10 & 18-20	240	120	90	Urea (46% N)
T4	Double-layer CRU	8-10 & 18-20	240	120	90	CRU (44% N)

### 2.2 Calculation of N_2_O emission flux and related indicators

The N_2_O measurements were taken using the manual closed static chamber method over two wheat-growing seasons from 2021-2023 (Deng et al., 2019). The chamber system consisted of two components: a base collar and a chamber cover. The base collar was inserted 15 cm below the soil surface in each tested unit, while the chamber cover (10 cm width × 20 cm length × 30 cm height) was placed on top of the base collar to facilitate gas sampling. The top edge of the base had a groove to seal the rim of the chamber by filling it with water. A 60 mL syringe was used to extract gas samples from the chamber, which were then stored in pre-evacuated 30 mL vacuum vials. Gas samples were collected at 0, 10, 20, and 30-minutes intervals immediately after sealing the chamber. A thermometer was placed inside the chamber to monitor temperature. To reduce the effects of daily fluctuations, gas samples were typically collected between 8:00 am and 10:30 am. The gas samples were collected during the initial three days after fertilization in the first week, every 2 days in the 2^nd^ and 3^rd^ weeks, and every 10 days from the 4^th^ week until maturity. The collected gas samples were analyzed within a week using a gas chromatograph equipped with both an electron capture detector (ECD) and a flame ionization detector (FID). Then, the N_2_O flux (F) was determined by utilizing the following Equation 1 provided by Liu et al. (2017).


(1) $$F=P\times \frac{V}{A}\times \frac{\Delta R}{\Delta t}\times \frac{273}{\left(273+T\right)}$$

Where F represents the emission flux of N_2_O, measured in μg m^−2^ h^−1^, ρ is the density of the gas in its standard state (kg m^−3^), V is the volume of the static chamber in cubic meters (m^3^), A represents the soil surface area covered by the chamber in square meters (m^2^), ΔR/Δt denotes the rate of change of N_2_O concentration within the chamber per unit time, measured in microliters per liter per minute (μL L^−1^ min^−1^), T is the mean temperature within the chamber in degrees Celsius. The computation of the cumulative N_2_O emission was carried out using the Equation 2, the methodology suggested by Ding et al. (2007).


(2) $$\begin{array}{c} Cumulative\;N_{2}O\;\left(kg\;ha^{-1}\right)\\ =\sum (\frac{F_{i+1}+F_{i}}{2})\times \left(B_{i+1}-B_{i}\right)\times \frac{24}{1000} \end{array}$$

Here, F_i_ and F_i+1_ as a function of the N_2_O fluxes (μg m^-2^ h^-1^) at two consecutive measurements, and B_i_ and B_i+1_ are the number of days between these measurements.

The procedure described by Rychel et al. (2020) was adopted to calculate the both N_2_O emission factor (%) and the yield-scaled N_2_O emission (kg t^-1^) in Equations 3, 4.


(3) $$\begin{array}{c} N_{2}O\;EF\;\left(\%\right)=\\ \frac{Cumulative\;N_{2}O\;fertilizer\;treatment-Cumulative\;N_{2}O\;control\;treatment}{N\;fertilizer\;amount}\times 100 \end{array}$$

(4) $$Yield\;scale\;N_{2}O\;emission\;\left(kg\;t^{-1}\right)=\frac{Cumulative\;N_{2}O}{Grain\;yield}$$

### 2.3 Evaluation of soil inorganic N content (NO_3_^−^-N, NH_4_^+^-N)

The soil samples were collected to examine the amounts of inorganic N during the different soil layers at the overwintering, jointing, anthesis and maturity stages. The soil samples were obtained vertically in each treatment with the help of a soil drill at depths of 0–10 cm, 10–20 cm, 20–30 cm and 30–40 cm. The freshly excavated soil was quickly transferred to the testing center and sieved through a 2-mm mesh screen. By the use of Seal Analytical AA3 HR Nutrient Autoanalyzer, the inorganic N (NO_3_ **^−^**-N, NH_4_^+^-N) amount was measured as described by Geng et al. (2016).

### 2.4 Evaluation of wheat grain yield and plant dry matter production

Grain yield was evaluated at maturity by randomly selecting a 1 m^2^ area from each plot, harvesting it and allowing it to dry naturally. The amount of moisture content was subsequently determined using a moisture analyzer and expressed at 14%. The plant dry matter was calculated at physiological maturity, twenty fully developed plants were taken consecutively from each treatment and cut at the base, divided into spikes, leaves and stem + leaf sheath. The samples were oven-dried at 72°C for 48 hours, after which the biomass was weighed. The spike was divided into two components: grain and spike axis + grain husk. The total plant dry matter was calculated by summing the dry matter of the spike, stem + leaf sheath and leaves. To determine the dry matter per hectare, the dry weight of each plant was multiplied by the total number of plants per hectare for each treatment.

### 2.5 Determination of N uptake amount in plant

The oven-dried samples were crushed into a fine powder and the N content of each part of the plants was determined using an elemental analyzer (Vario PYRO, Elementar, Germany), following the procedure described in (Deng et al., 2019; Ye et al., 2013).

### 2.6 Determination of N use efficiency and related parameters

The N uptake efficiency (NUpE) and N recovery efficiency (NRE) were determined using the N uptake data in Equations 5, 6. N use efficiency (NUE) and partial factor productivity (PFPN) were determined by the agronomic approach in Equations 7, 8, adopting the methodology described by Moll et al. (1982) and Ye et al. (2013).


(5) $$NUpE\;\left(kg\;kg^{-1}\right)=\;\frac{Total\;N\;uptake}{N\;application\;amount}$$

(6) $$\begin{array}{c} NRE\;(kg\;kg^{-1})=\\ \frac{N\;uptake\;from\;N\;treatment-N\;uptake\;from\;control\;treatment}{N\;application\;amount}\times 100 \end{array}$$

(7) $$\begin{array}{c} NUE(kg\;kg^{-1})=\\ \frac{Grain\;production\;\left(N\;treatment\right)-Grain\;production\;\left(control\;treatment\right)}{N\;application\;amount} \end{array}$$

(8) $$PFPN(kg\;kg^{-1})=\frac{Grain\;production}{N\;application\;amount}$$

### 2.7 Statistical analysis

The differences between the various treatments were analyzed using an analysis of variance, computed with SPSS (version 21.0). The LSD test, at a significance level of 0.05, was utilized to examine the treatment means. The reported determinations were the average values obtained from three replicates. The figures were generated using Origin Pro 21. We used the R package “lavaan” (Rosseel, 2012) for structural equation modeling (SEM) to evaluate the interactions among N_2_O emissions, grain yield, NO_3_^−^-N, NH_4_^+^-N, soil moisture, N uptake and soil temperature, under the effects of different N fertilizer types and layered fertilization strategies.

## 3 Results

### 3.1 Soil temperature and moisture in different soil layers

Soil temperature increased rapidly after the sowing of winter wheat and then decreased in winter. Across all treatments, the temperature at 20 cm soil depth was consistently higher and exhibited smaller daily fluctuations as compared to 10 cm. Compared to the T1, T2 reduced the average annual soil temperature by 1.83% at 10 cm and 2.65% at 20 cm soil depth. A similar trend was observed in T4, which showed a 2.83% reduction in average annual soil temperature at 10 cm and 3.65% at 20 cm soil depth relative to T3. Furthermore, T3 resulted in a 2.75% reduction in average annual soil temperature at 10 cm and 3.58% at 20 cm soil depth as compared to T1. Compared to the T2, T4 resulted in a 3.74% reduction in average annual soil temperature at 10 cm and 4.55% at 20 cm soil depth (**Figure 2**).

Across all treatments, soil moisture at 20 cm soil depth was consistently optimized and exhibited smaller daily fluctuations relative to 10 cm. The results indicated that compared to the T1, T2 increased average annual soil moisture by 8.13% at 10 cm and 8.85% at 20 cm soil depth. A similar trend was observed in T4, which showed a 12.31% higher average annual soil moisture at 10 cm and 12.54% at 20 cm soil depth as compared to T3. Furthermore, T3 resulted in a 6.07% higher average annual soil moisture at 10 cm and 6.30% at 20 cm soil depth as compared to T1. Compared to the T2, T4 resulted in a 10.17% higher average annual soil moisture at 10 cm soil depth and 9.91% at 20 cm soil depth (**Figure 3**). Our findings indicated that low soil temperature and optimized moisture were observed in the double-layer fertilization of CRU.

### 3.2 Gaseous N_2_O emission

**Figure 4:**
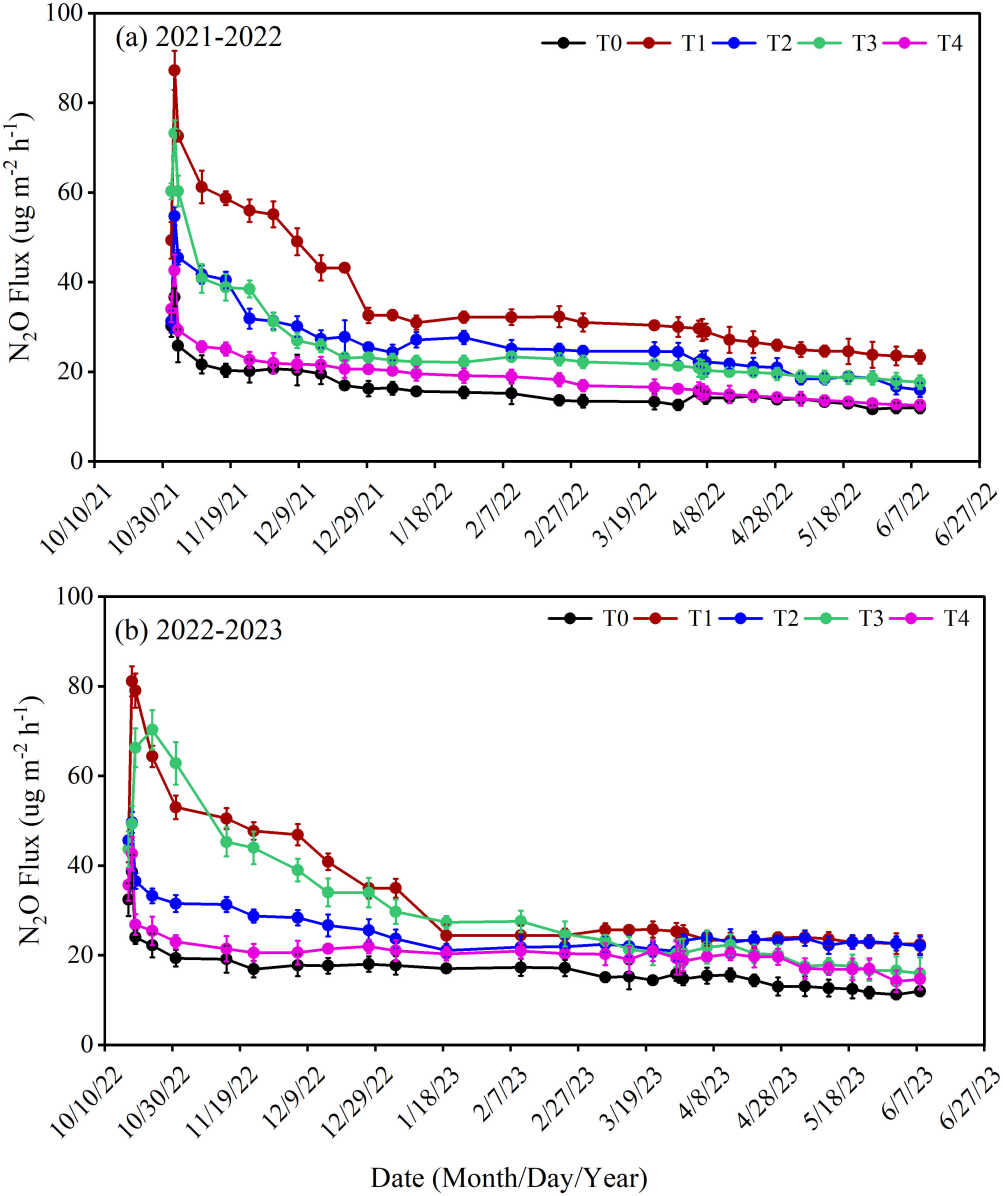
Surface N_2_O flux (μg m^-2^ h^-1^) during winter wheat development under various treatments from 2021-2022 **(a)**, 2022-2023 **(b)**. The differences between treatments were analyzed using analysis of variance (ANOVA), and the least significant difference (LSD) test at a significance level of 0.05 was applied to compare the treatment means.

The dynamics of N_2_O flux were affected by the different types of N fertilizer and the method of layered fertilization between two winter wheat-growing periods from 2021-2023 (*p< 0.05*). The fluctuations in N_2_O emissions during crop growth are demonstrated in **Figure 4**. During these two seasons, a significant elevation in soil N_2_O flux was observed within one week of fertilization. Subsequently, the emission flux of N_2_O gradually decreased. The highest peak N_2_O emission flux of 87.25 μg m^-2^ h^-1^ for T1 and 73.19 μg m^-2^ h^-1^ for T3 treatment were observed in 2021-2022. The T4 and T2 treatments displayed minimal N_2_O emission fluxes, reaching a maximum peak of 42.51 μg m^-2^ h^-1^ and 54.70 μg m^-2^ h^-1^. A similar pattern was observed in the second wheat growing season, both treatments T1 and T3 showed high peaks of N emissions, with T1 reaching 81.12 μg m^-2^ h^-1^ and T3 reaching 70.28 μg m^-2^ h^-1^. The T4 and T2 treatments exhibited a relatively low peak in N_2_O flux, measuring 42.95 μg m^-2^ h^-1^ and 49.66 μg m^-2^ h^-1^, respectively. The T4 treatment consistently exhibited minimal N_2_O emission flux during both growing seasons.

**Figure 5:**
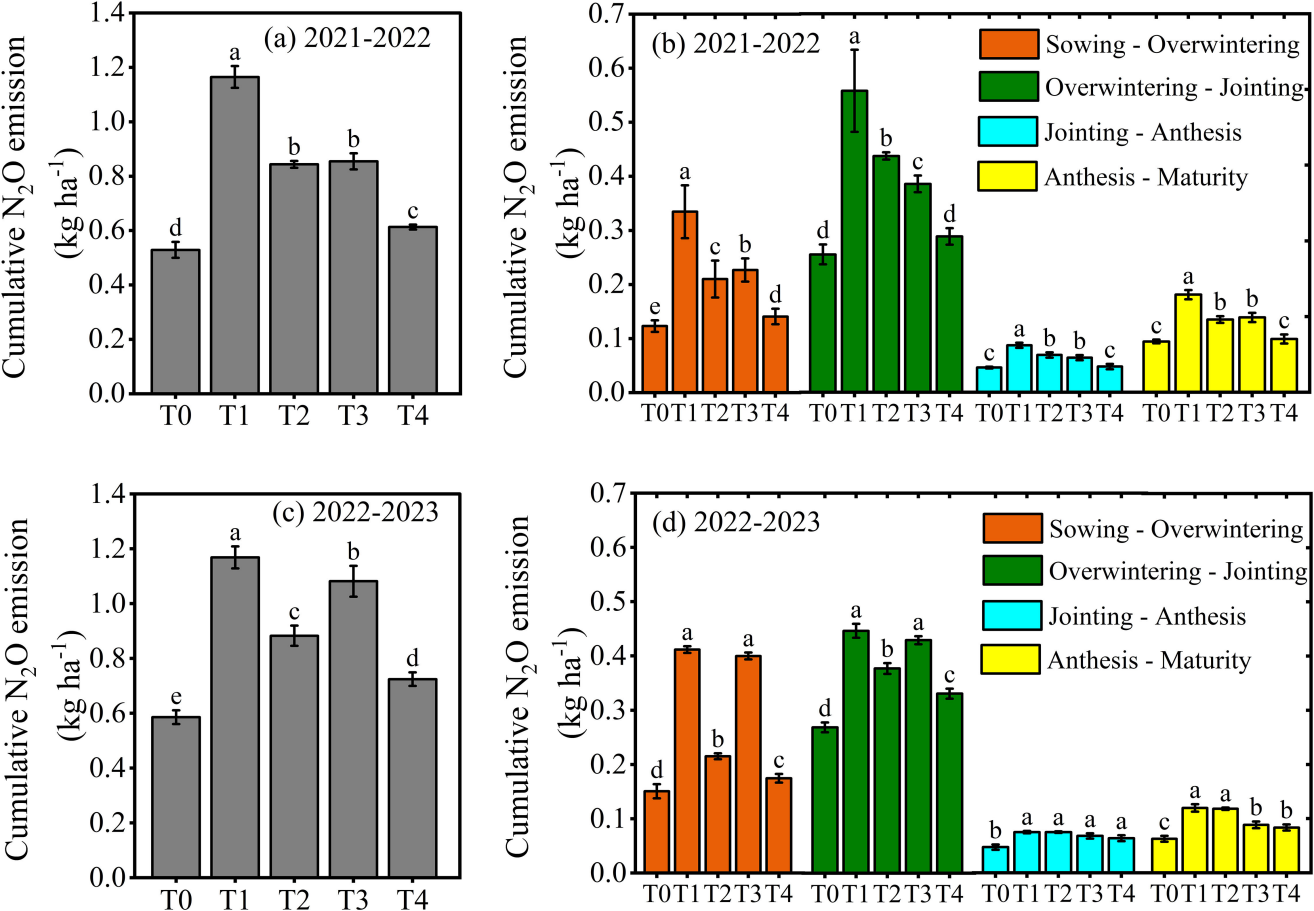
Cumulative N_2_O emissions across various growth stages **(b, d)** and the entire growth period **(a, c)** of winter wheat (2021–2023) in response to layered applications of different nitrogen fertilizers. The differences between treatments were analyzed using analysis of variance (ANOVA), and the least significant difference (LSD) test at a significance level of 0.05 was applied to compare the treatment means.

N_2_O emissions during different wheat growth stages were affected by the layering placement of different N fertilizers during the period from 2021-2023 (**Figure 5**, *p< 0.01*). Soil N_2_O emissions increased markedly from sowing-jointing stage, followed by a subsequent decline. Compared to T1, T2 reduced the N_2_O emission by 41.35% during the sowing-overwintering, 18.49% during the overwintering-jointing, 17.36% during the jointing-anthesis and 23.07% during the anthesis-maturity stage. The same trend was observed in T4, which reduced the N_2_O emission by 48.31% during the sowing-overwintering, 24.44% during the overwintering-jointing, 15.47% during the jointing-anthesis and 20.55% during the anthesis-maturity stage as compared to T3. Furthermore, compared to T1, T3 resulted in a 16.36% reduction in N_2_O emission during the sowing-overwintering, 17.70% during overwintering-jointing, 45.83% during jointing-anthesis and 24.03% during the anthesis-maturity stage. The T4 exhibited a reduction of 28.02% N_2_O during the sowing-overwintering, 23.62% during overwintering-jointing, 21.27% during jointing-anthesis and 31.66% during the anthesis-maturity stage as compared to T2.

**Figure 6:**
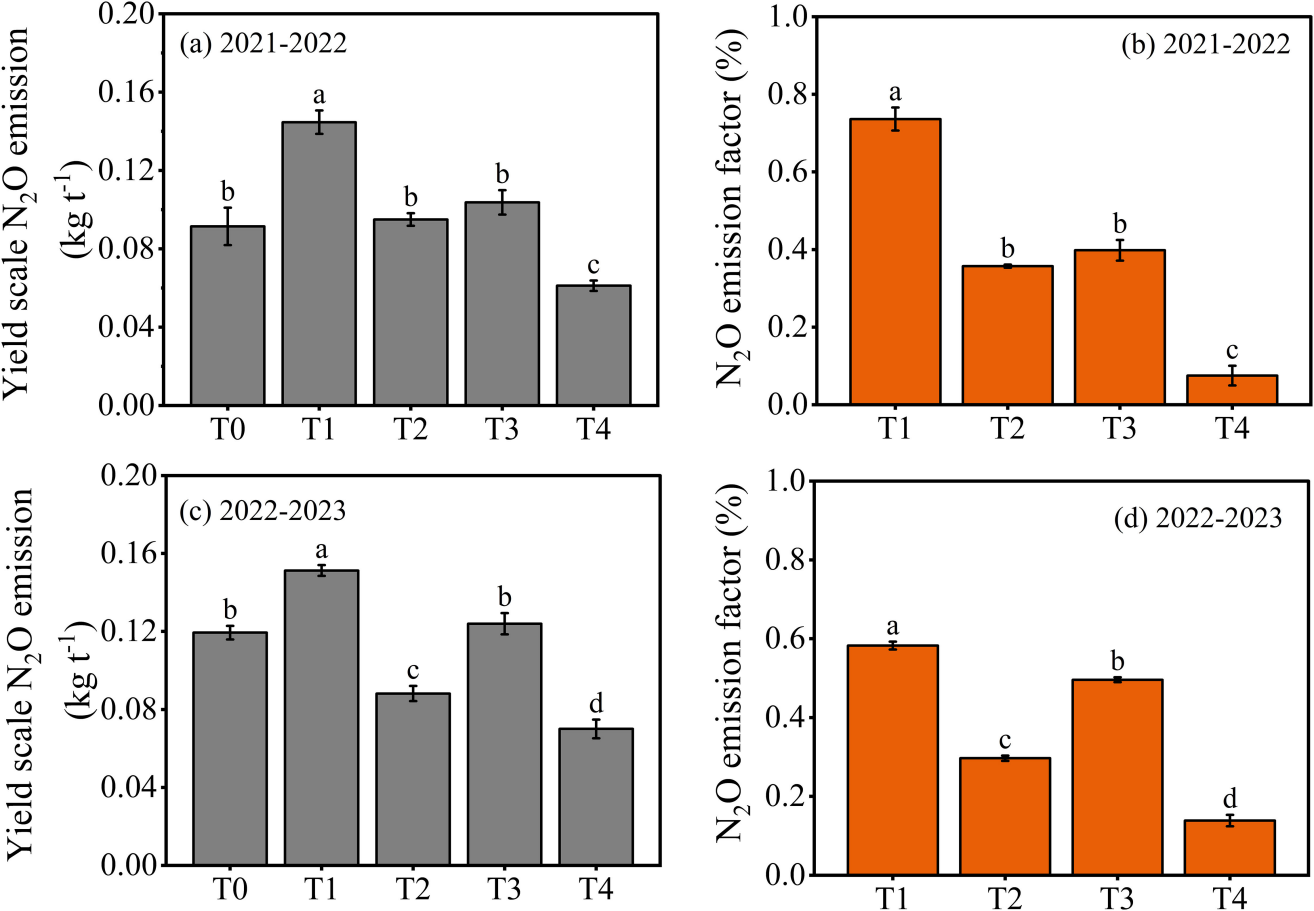
Yield scale N_2_O emission **(a, c)** and emission factor **(b, d)** responses to layering application of different N fertilizers in winter wheat from 2021-2023. The differences between treatments were analyzed using analysis of variance (ANOVA), and the least significant difference (LSD) test at a significance level of 0.05 was applied to compare the treatment means.

The cumulative N_2_O emissions, yield scale N_2_O emission and emission factor were influenced by both the types and layering application of different N fertilizers (**Figures 5**, **6**). The T2 resulted in a 26.03% reduction of cumulative N_2_O emissions, 37.85% in yield scale N_2_O emission and 49.81% in N_2_O emission factor compared to T1. The same trend was observed in T4, which showed a 30.94% reduction of cumulative N_2_O emissions, 40.83% in yield scale N_2_O emission and 76.00% in N_2_O emission factor compared to T3. Furthermore, compared to T1, T3 resulted in a 17.01% reduction of cumulative N_2_O emissions, 24.28% in yield scale N_2_O emission and 29.86% in N_2_O emission factor. The T4 resulted in a 22.52% reduction of cumulative N_2_O emissions, 27.78% in yield scale N_2_O emission and 65.55% in N_2_O emission factor compared to T2. The results indicated that single-layer fertilization of urea produced more N_2_O during all growth stages than double-layer fertilization of CRU.

### 3.3 Soil inorganic N content in different soil layers

#### 3.3.1 Ammonium N content

**Figure 7:**
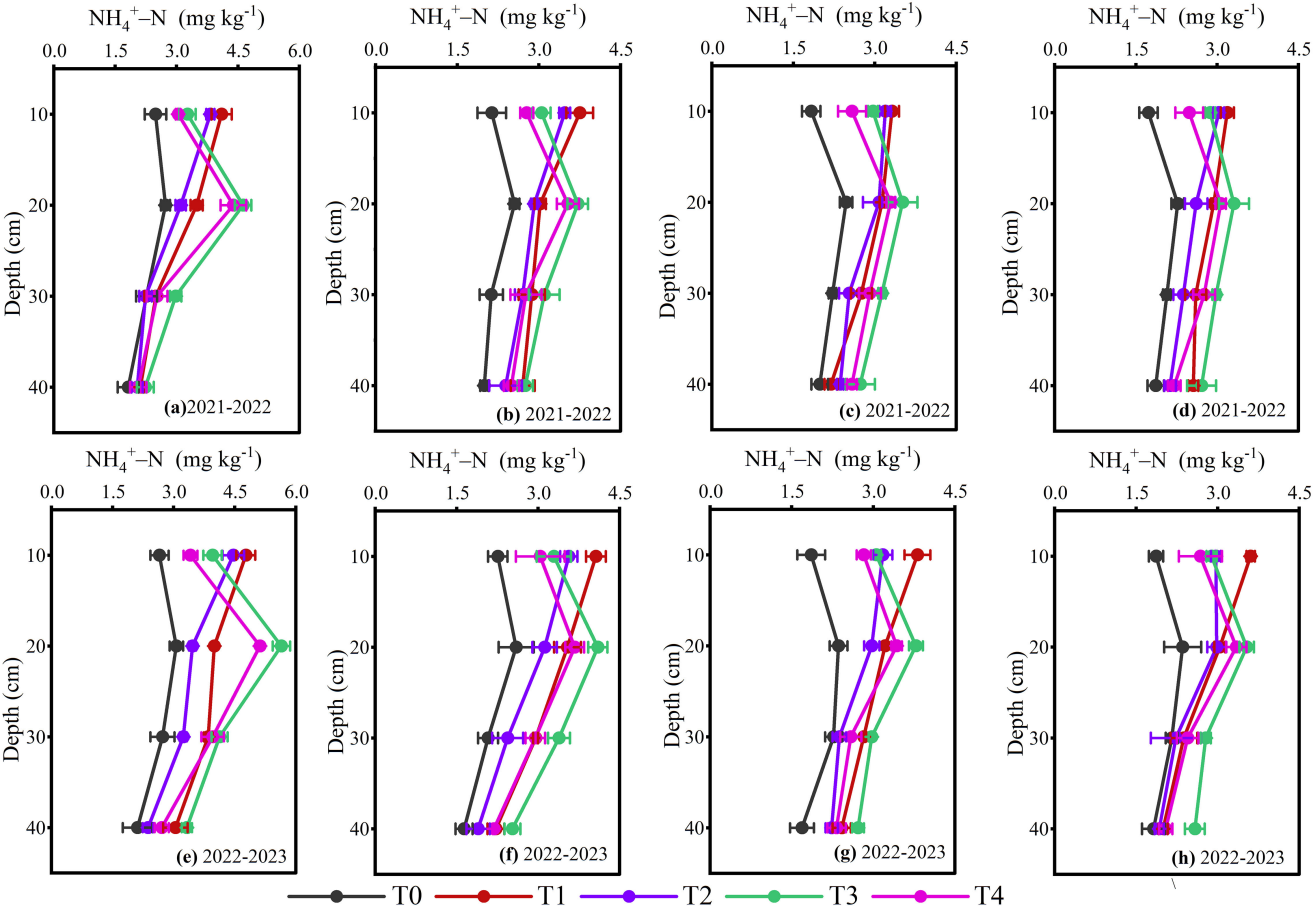
NH_4_^+^-N content responded to the layering application of different N fertilizers at overwintering **(a, e)**, jointing **(b, f)**, anthesis **(c, g)** and maturity **(d, h)**. The differences between treatments were analyzed using analysis of variance (ANOVA), and the least significant difference (LSD) test at a significance level of 0.05 was applied to compare the treatment means.

The ammonium N content (NH_4_^+^-N) in 0–40 cm various soil layers were influenced by the layered placement of different N fertilizers during different wheat growth periods in 2021-2023 (**Figure 7**; *p< 0.05*). The two-year average results demonstrated that during the overwintering stage, a higher concentration of NH_4_^+^-N was detected at a soil depth of 0–10 cm under single-layer fertilization. In contrast, under double-layer fertilization, the highest concentration was observed at a soil depth of 10–20 cm, followed by a gradual decrease in concentration throughout the wheat development. Compared to conventional urea treatments (T1 and T3), CRU treatments (T2 and T4) reduced NH_4_^+^-N content in the 0–10 cm soil depth by 6.56% and 10.65% during the overwintering, 9.88% and 8.16% at jointing, 10.68% and 10.18% at anthesis, 11.66% and 10.27% at the maturity stage, respectively. The double-layer approaches (T3 and T4) resulted 18.59% and 22.16% reduction in NH_4_^+^-N content in 0–10 cm soil depth during the overwintering, 18.87% and 17.32% at the jointing, 15.54% and 15.06% at the anthesis, 15.15% and 13.82% at the maturity stage compared to single-layer (T1 and T2), respectively. The overall trend of two-year average NH_4_^+^-N at 0–10 cm (T1>T2, T3>T4, T1>T3 and T2>T4) and 10–20 cm soil layers (T1>T2, T3>T4, T3>T1 and T4>T2) was present.

#### 3.3.2 Nitrate N content

**Figure 8:**
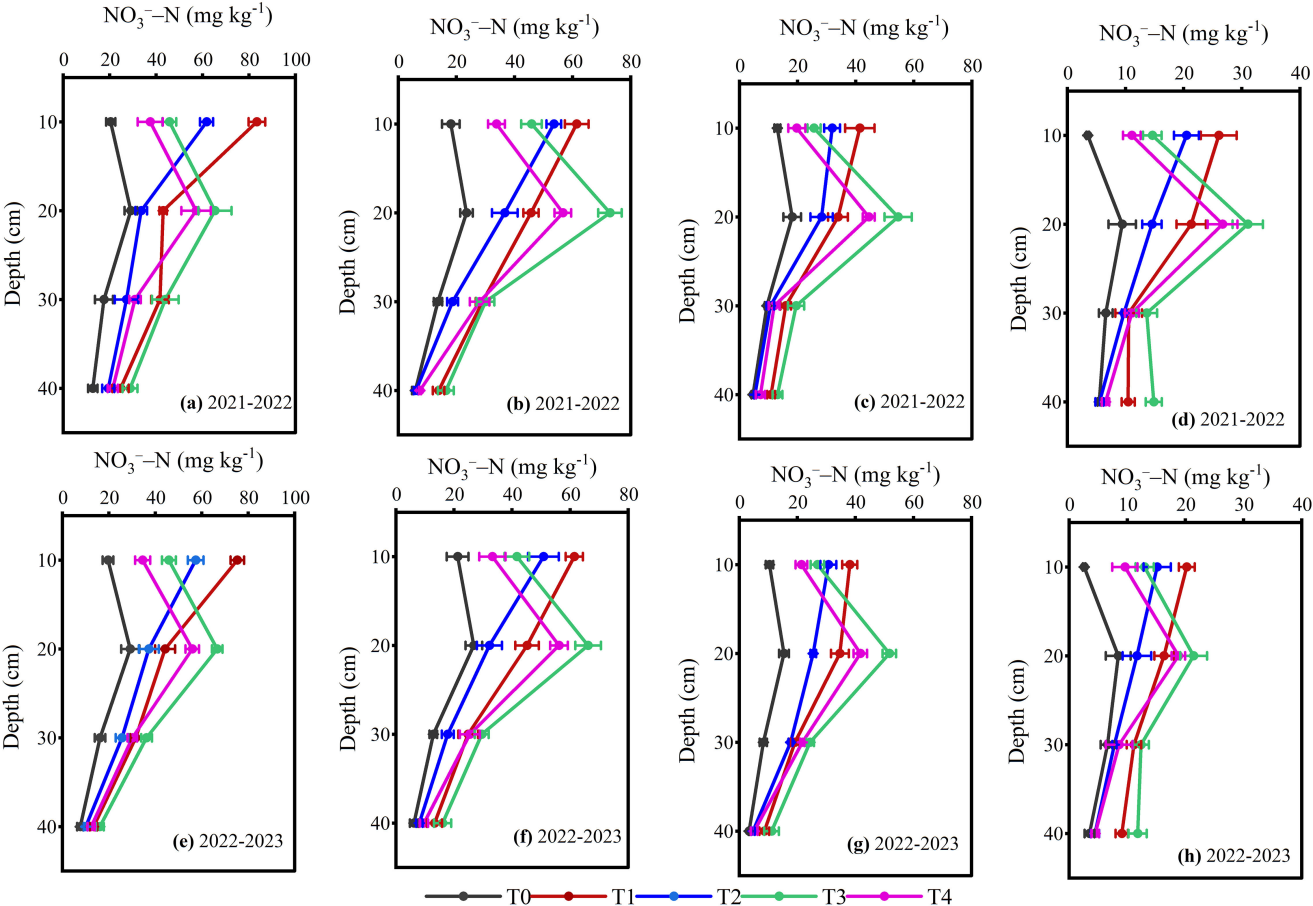
NO_3_^–^ –N content responded to the layering application of different N fertilizers at overwintering **(a, e)**, jointing **(b, f)**, anthesis **(c, g)** and maturity **(d, h)**. The differences between treatments were analyzed using analysis of variance (ANOVA), and the least significant difference (LSD) test at a significance level of 0.05 was applied to compare the treatment means.

The impact of layered placement of different N fertilizers on soil nitrate N content (NO_3_^−^-N) at 0–40 cm different soil layers were discovered to be statistically significant across all growth stages 2021-2023 (**Figure 8**; *p< 0.05*). The two-year average results indicated that during the overwintering stage, a higher level of NO_3_^−^-N was detected in the 0–10 cm soil layer under the one-time single-layer fertilization. In contrast, double-layer fertilization showed higher NO_3_^−^-N concentration at 10–20 cm soil depth, which gradually declined as wheat development progressed. Compared to T1 and T3, T2 and T4 resulted in a 24.94% and 21.15% reduction NO_3_^−^-N content in 0–10 cm soil depth during the overwintering, 12.96% and 25.78% at jointing, 25.02% and 23.09% at anthesis, 21.90% and 24.24% at the maturity stage of winter wheat, respectively. Furthermore, the double-layered approaches (T3 and T4) resulted in a 42.73% and 39.84 more reduction in NO_3_^−^–N content at 0–10 cm soil depth during the overwintering, 26.26% and 37.13% at jointing, 38.76% and 37.18% at anthesis, 44.42% and 46.08% at the maturity stage compared to conventional urea (T1 and T2), respectively. The overall trend of two-year average NO_3_^−^-N at 0–10 cm (T1>T2, T3>T4, T1>T3 and T2>T4) and 10–20 cm soil layers (T1>T2, T3>T4, T3>T1 and T4>T2) was present. Our study findings suggest that double-layer fertilization of CRU resulted in a low concentration of NO_3_^−^-N in the top 0–10 cm soil depth, while higher at 10–20 cm soil depth and remained relatively stable.

### 3.4 Crop N uptake

The layering placement of different N fertilizers had a significant influence on the N uptake of winter wheat plants from 2021-2023 (**Table 2**; *p< 0.05*). Single-layer CRU (T2) led to an increased 21.05% N uptake per area in stem + sheath, 30.64% in leaf, 34.95% in spike axis + grain husk, 25.07% in grain, and 28.06% in the total plant compared to T1. Similarly, double-layer CRU (T4) showed a 19.59% further increase in N uptake in the stem + sheath, 35.81% in the leaf, 28.10% in the spike axis + grain husk, 14.77% in the grain, and 28.06% in total plant relative to T3 at the maturity stage. Furthermore, T3 resulted in a significant enhancement in N uptake by 10.62% in the stem + sheath, 18.36% in the leaf, 18.98% in the spike axis + grain husk, 21.36% in the grain, and 19.09% in the total plant relative to T1. The T4 resulted in a significant enhancement in N uptake by 9.42% in the stem + sheath, 21.71% in the leaf, 12.99% in the spike axis + grain husk, 8.08% in the grain, and 9.48% in the total plant as compared to T2. These results indicate that CRU enhanced N uptake in winter wheat, particularly when applied in a double-layer configuration.

**Table 2:** Crop N uptake at maturity stage responses to layering application of different N fertilizers in winter wheat from 2021-2023.

Growing year	Treatments	Nitrogen uptake (kg ha^-1^)				
		Stem + Sheath	Leaf	Spike axis + Grain husk	Grain	Total plant
2021-2022	T0	27.2 ± 0.9d	12.3 ± 0.3c	11.0 ± 0.6d	80.5 ± 2.8e	131.0 ± 4.6e
	T1	37.1 ± 0.6c	15.9 ± 1.4bc	13.8 ± 1.1cd	145.0 ± 2.8d	211.8 ± 2.4d
	T2	43.8 ± 1.4ab	20.0 ± 0.8ab	17.7 ± 1.0ab	191.0 ± 4.3b	272.5 ± 7.6b
	T3	39.3 ± 1.7bc	18.6 ± 1.0b	16.3 ± 0.5bc	177.4 ± 1.6c	251.6 ± 4.3c
	T4	46.5 ± 1.6a	24.4 ± 2.4a	20.5 ± 2.6a	204.7 ± 2.3a	296.1 ± 3.9a
2022-2023	T0	23.7 ± 3.5b	11.5 ± 0.9c	9.8 ± 1.0d	67.8 ± 3.2c	112.8 ± 4.9c
	T1	31.1 ± 4.3ab	14.4 ± 2.5bc	12.6 ± 0.7cd	154.1 ± 9.1b	212.2 ± 6.9b
	T2	38.5 ± 3.6a	19.6 ± 1.2ab	17.8 ± 1.2ab	194.4 ± 2.0ab	270.3 ± 1.6ab
	T3	35.8 ± 4.0ab	16.9 ± 1.8bc	15.1 ± 1.6bc	185.5 ± 23.3ab	253.3 ± 29.7ab
	T4	43.3 ± 4.2a	23.8 ± 1.8a	19.6 ± 2.4a	211.7 ± 20.0a	298.4 ± 32.8a

T0 = no nitrogen fertilizer, T1 = one-time single-layer fertilization of urea at 8–10 cm depth, T2 = one-time single-layer fertilization of CRU at 8–10 cm depth, T3 = one-time double-layer fertilization of urea at 8–10 and 18–20 cm depth, and T4 = one-time double layer fertilization of CRU at 8–10 and 18–20 cm depth. The results in all sections illustrate the average of 3 replicates for each treatment with standard errors. The differences between treatments were analyzed using analysis of variance (ANOVA), and the least significant difference (LSD) test at a significance level of 0.05 was applied to compare the treatment means.

### 3.5 Dry matter production

Layering placement of different N fertilizers had a significant effect on the dry matter production of winter wheat from 2021-2023 (**Table 3**; *p< 0.05*). Single-layer CRU (T2) produced 14.33% more dry matter in the stem + sheath, 17.02% in the leaf, 13.13% in the spike axis + grain husk, 11.93% in the grain, and 13.64% in the total compared to T1. Similarly, double-layer CRU (T4) demonstrated 10.81% higher dry matter in the stem + sheath, 16.15% in the leaf, 16.47% in the spike axis + grain husk, 10.69% in the grain, and 12.10% in the total as compared to T3. Furthermore, when compared to T1, the T3 exhibited a significantly increased dry matter production by 8.83% in the stem + sheath, 7.10% in the leaf, 7.21% in the spike axis + grain husk, 6.23% in the grain, and 7.31% in the total plant. The double-layer approach (T4) exhibited a significantly increased dry matter production by 5.49% in the stem + sheath, 6.30% in the leaf, 10.37% in the spike axis + grain husk, 5.05% in the grain, and 5.86% in the total plant compared to single-layer (T2).

**Table 3:** Crop dry matter production at maturity stage responses to layering application of different N fertilizers in winter wheat from 2021-2023.

Growing year	Treatments	Dry matter (t ha^-1^)				
		Stem + Sheath	Leaf	Spike axis + Grain husk	Grain	Total plant
2021-2022	T0	4.8 ± 0.2c	2.3 ± 0.4c	1.3 ± 0.1c	5.1 ± 0.8e	13.5 ± 2.6d
	T1	6.1 ± 0.7b	3.1 ± 0.6b	1.7 ± 0.1b	7.8 ± 1.3d	18.7 ± 1.4c
	T2	6.9 ± 0.9a	3.7 ± 0.2ab	1.9 ± 0.4ab	8.9 ± 1.1b	21.3 ± 3.2b
	T3	6.8 ± 0.9ab	3.3 ± 0.3b	1.8 ± 0.2b	8.3 ± 0.9c	20.1 ± 2.9b
	T4	7.4 ± 1.0a	4.0 ± 0.8a	2.1 ± 0.6a	9.4 ± 1.9a	22.8 ± 3.0a
2022-2023	T0	4.0 ± 0.3b	2.2 ± 0.2b	1.2 ± 0.1c	4.6 ± 1.1b	11.9 ± 2.1b
	T1	6.2 ± 1.4a	2.9 ± 0.6ab	1.5 ± 0.3b	8.3 ± 1.6a	19.0 ± 2.9a
	T2	7.2 ± 0.9a	3.4 ± 0.9a	1.8 ± 0.5a	9.1 ± 1.2a	21.5 ± 3.2a
	T3	6.7 ± 1.1a	3.2 ± 0.4a	1.7 ± 0.1ab	8.7 ± 0.9a	20.3 ± 1.4a
	T4	7.5 ± 1.8a	3.6 ± 0.2a	2.0 ± 0.7a	9.4 ± 0.8a	22.5 ± 1.8a

The results in all sections illustrate the average of 3 replicates for each treatment with standard errors. The differences between treatments were analyzed using analysis of variance (ANOVA), and the least significant difference (LSD) test at a significance level of 0.05 was applied to compare the treatment means.

### 3.6 Grain yield

The grain yield, productive spike number, grains per spike and thousand-grain weight of winter wheat from 2021–2023 were affected by the layering placement of different N fertilizers (**Table 4**; *p< 0.05*). The two-year average results indicated that single-layer CRU treatment (T2) increased grain yield, productive spike number, grains per spike, and thousand-grain weight by 20.56%, 19.72%, 3.23% 7.76% respectively compared to T1. Similarly, T4 increased grain yield, productive spike number, grains per spike, and thousand-grain weight by 14.74%, 8.72%, 3.11% and 9.48% respectively compared to T3. Furthermore, double-layer (T3) resulted an increased grain yield, productive spike number, grains per spike and thousand-grain weight by 11.31%, 16.37%, 1.74%, and 3.52% respectively, compared to T1. Compared to T2, double-layer CRU (T4) treatment further improved grain yield, productive spike number, grains per spike, and thousand-grain weight by 5.94%, 5.57%, 1.62%, and 5.16% respectively.

**Table 4:** Grain yield, spikes, grain per spike, and 1000 grain weight (GW) responses to layering application of different N fertilizers in winter wheat from 2021-2023.

Growing year	Treatments	Grain yield (t ha^-1^)	Spikes (×10^4^ ha ^-1^)	Grain per spike	1000 GW (g)
2021-2022	T0	5.8 ± 0.3c	430.7 ± 10.3c	34.7 ± 1.9b	33.7 ± 1.6b
	T1	8.2 ± 0.9b	478.7 ± 10.5b	43.5 ± 1.8a	38.4 ± 1.2a
	T2	9.2 ± 1.2ab	620.3 ± 15.6a	44.7 ± 0.9a	40.7 ± 1.3a
	T3	9.0 ± 1.6ab	608.7 ± 9.2a	43.7 ± 2.1a	39.5 ± 1.4a
	T4	9.9 ± 1.9a	648.9 ± 15.3a	45.1 ± 1.1a	41.8 ± 1.7a
2022-2023	T0	4.9 ± 0.3d	380.3 ± 11.5d	36.2 ± 1.8b	31.2 ± 1.1c
	T1	7.7 ± 1.4c	515.9 ± 13.1c	43.8 ± 1.0a	37.7 ± 1.5b
	T2	10.0 ± 1.3ab	566.7 ± 15.0ab	45.4 ± 1.1a	41.4 ± 0.8ab
	T3	8.8 ± 0.9bc	544.7 ± 11.0bc	45.1 ± 0.8a	39.3 ± 1.1b
	T4	10.5 ± 0.8a	603.7 ± 11.5a	46.4 ± 0.9a	44.5 ± 1.0a

The results in all sections illustrate the average of 3 replicates for each treatment with standard errors. The differences between treatments were analyzed using analysis of variance (ANOVA), and the least significant difference (LSD) test at a significance level of 0.05 was applied to compare the treatment means.

### 3.7 Nitrogen use efficiency

The different types of N fertilizer and layering placement have significant impact on N uptake efficiency (NUpE), N recovery efficiency (NRE), N use efficiency (NUE), and partial factor productivity of N (PFPN) from 2021-2023 (**Table 5**; *p< 0.05*). The T2 increased NUpE, NRE, NUE and PFPN by 27.12%, 68.79%, 31.66%, and 11.85% respectively compared to T1. The same trend was observed in T4, which increased NUpE, NRE, NUE and PFPN by 17.62%, 34.11%, 25.82%, and 10.75% respectively compared to T3. Furthermore, when compared to T1, T3 exhibited an increase in NUpE, NRE, NUE and PFPN by 18.64%, 47.30%, 16.40%, and 11.36% respectively. The T4 treatment increased NUpE, NRE, NUE and PFPN by 9.78%, 17.08%, 11.26% and 6.03% respectively compared to T2. The highest NUE was observed in the double-layer fertilization of CRU, attributed to enhanced N uptake and recovery efficiency of plants.

**Table 5:** Fertilizer use efficiency of nitrogen uptake (NUpE), N recovery (NRE), N use efficiency (NUE), and partial factor productivity (PFPN) responses to layering application of different N fertilizers in 2021-2023.

Growing year	Treatments	NUpE (kg kg^-1^)	NRE (kg kg^-1^)	NUE (kg kg^-1^)	PFPN (kg kg^-1^)
2021-2022	T0	—	—	—	—
	T1	0.88 ± 0.01d	33.66 ± 0.70d	11.05 ± 0.29d	34.20 ± 1.49b
	T2	1.13 ± 0.02b	58.95 ± 2.57b	15.64 ± 0.86ab	38.19 ± 1.13ab
	T3	1.04 ± 0.01bc	50.23 ± 0.20c	13.34 ± 0.38ac	37.41 ± 1.23ab
	T4	1.23 ± 0.01a	68.75 ± 1.35a	17.92 ± 0.10a	41.27 ± 1.78a
2022-2023	T0	—	—	—	—
	T1	0.89 ± 0.02b	41.11 ± 1.46b	15.46 ± 0.37a	32.20 ± 1.02b
	T2	1.12 ± 0.01ab	66.79 ± 0.39ab	18.83 ± 1.65a	41.86 ± 1.69a
	T3	1.06 ± 0.10ab	59.77 ± 10.11ab	17.33 ± 3.20a	36.50 ± 1.76ab
	T4	1.24 ± 0.11a	78.51 ± 11.15a	20.33 ± 3.16a	43.54 ± 3.47a

The results in all sections illustrate the average of 3 replicates for each treatment with standard errors. The differences between treatments were analyzed using analysis of variance (ANOVA), and the least significant difference (LSD) test at a significance level of 0.05 was applied to compare the treatment means.

### 3.8 Correlation analysis

**Figure 9:**
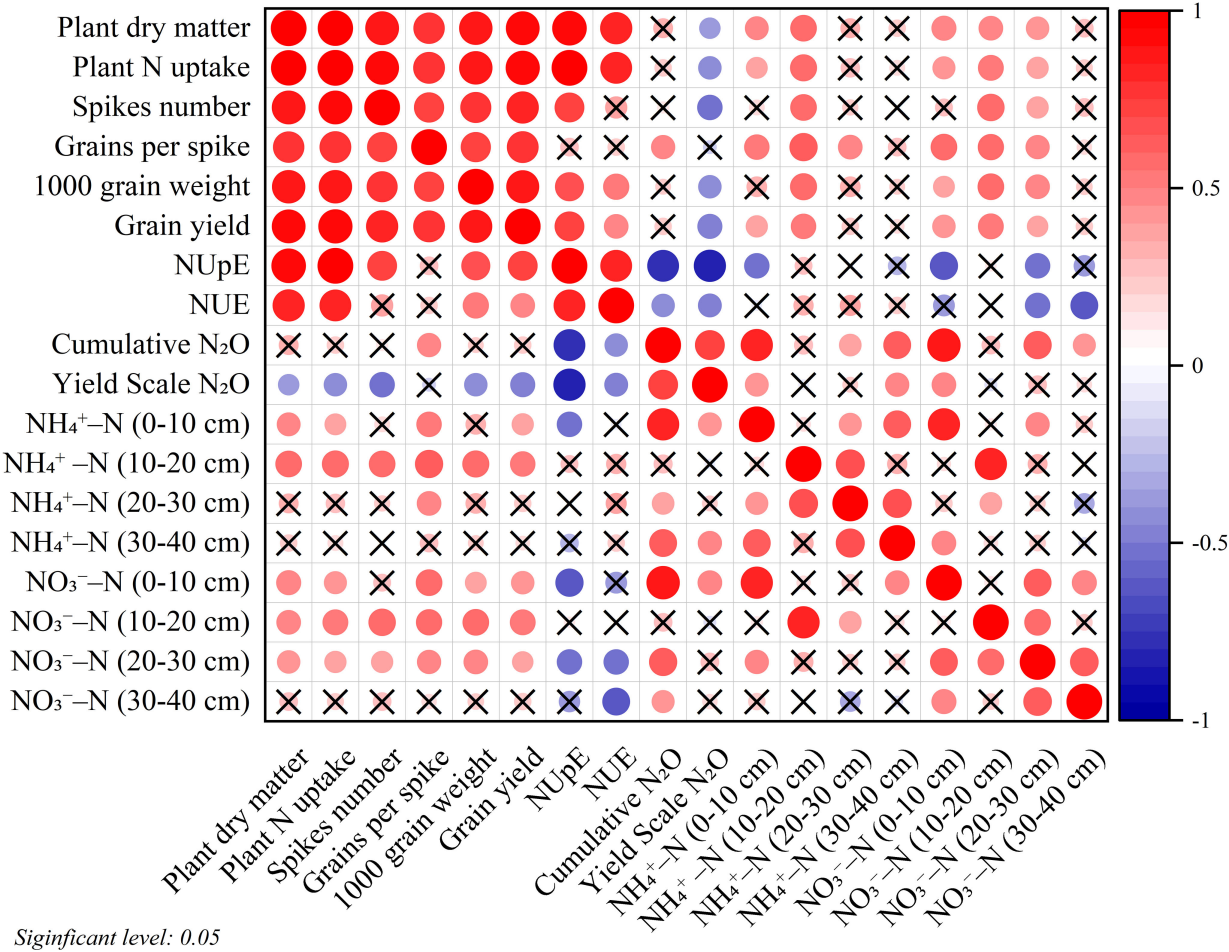
Pearson correlations analysis. Positive and negative associations are represented in red and blue, respectively. The graph’s larger and dark colors circle represent a stronger correlation and non-significance correlations are indicated by crosses (×).

The correlation analysis was conducted to examine the relationship between crop productivity, N uptake, N use efficiency, gaseous N_2_O emissions, and soil inorganic N content (**Figure 9**). A strongly significant and positive correlation was present between grain yield and several parameters, such as plant dry matter, plant N uptake, spikes number, N use efficiency, N uptake efficiency, and soil inorganic N content (0–20 cm soil depth). Conversely, a strong and negative correlation were present between yield scale N_2_O emission and parameters such as plant dry matter, plant N uptake, NUE, spikes number, and grain yield.

## 4 Discussion

**Figure 10:**
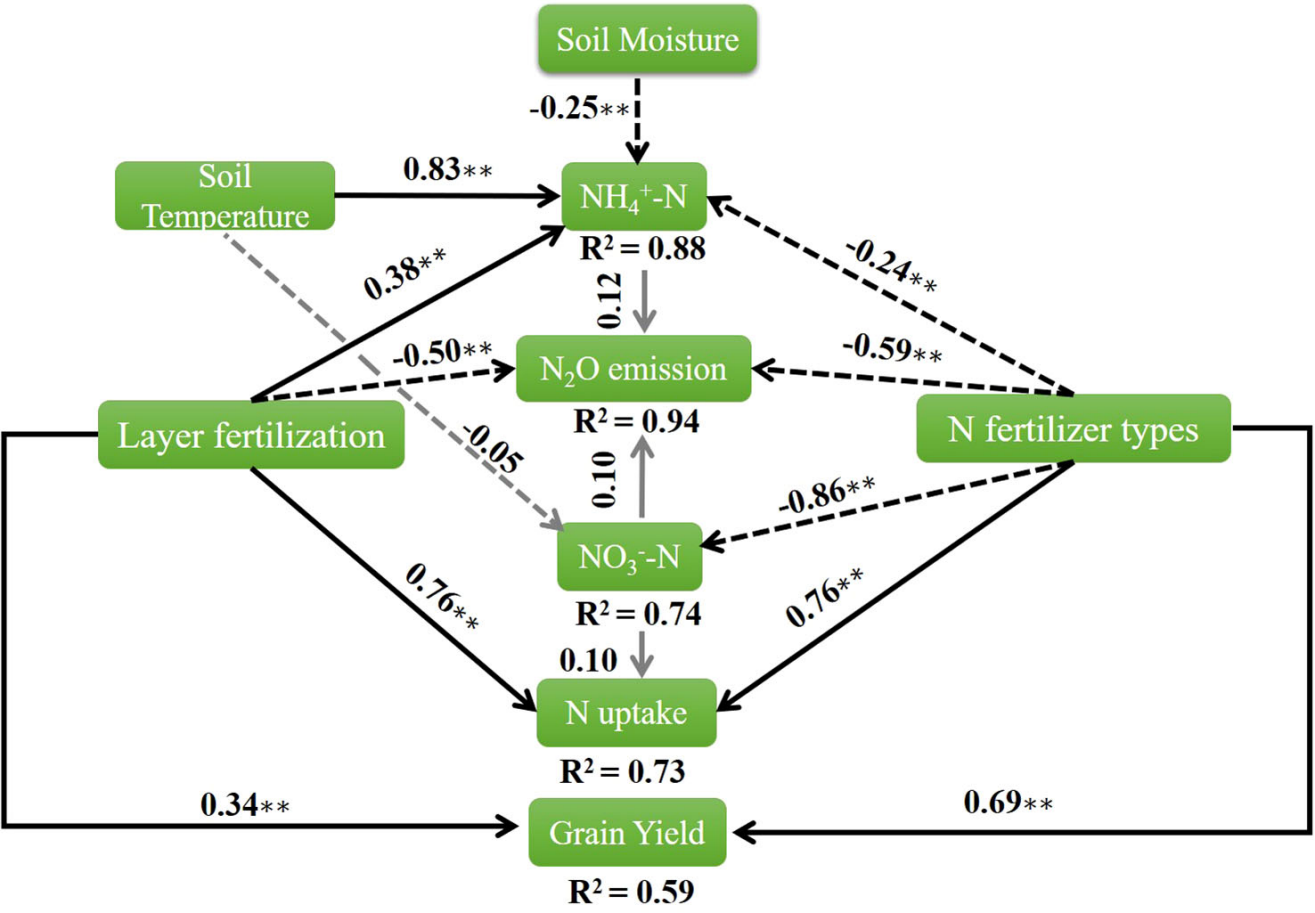
The results of structural equation modeling identified relationships among N_2_O emissions, grain yield, NO_3_^-^–N, NH_4_^+^–N, soil moisture, N uptake, and soil temperature under N fertilizer types and layered fertilization (P = 0.77, CFI = 1.00). Solid black arrows indicate the positive relationship, dotted black arrows indicate the negative relationship and the gray arrow indicates an insignificant relationship. The numbers adjacent to the arrows denote standardized coefficients, with significance levels marked by asterisks (*p< 0.05, **p< 0.001). The numbers near the boxes indicate the proportion of variance explained by the model (R^2^).

The depths of application of N fertilizer are an effective approach to affect crop grain yield. Single-layer N fertilization at 10–15 cm soil depth maximizes grain yield and dry matter production (Chen et al., 2023; Wu et al., 2021). However, some previous studies on the single-layer application of N fertilizers indicate that excessive depth of fertilization (25–35 cm) can have a negative impact on grain yield and dry matter (Chen et al., 2023; Huda et al., 2016; Wang et al., 2023; Wu et al., 2021). The reason for not increasing the yield may be the intermittent changes in root distribution during crop growth and the excessive application of N fertilizers to deep soil layers reduces N availability to crops during the seedling stage (Chen et al., 2022; Liu et al., 2022c). The study by Liu et al. (2024a) found that the highest grain yield of wheat was attained by the application of CRU at various soil depths. In our study, we observed that one-time double-layer fertilization of CRU (T4) increased wheat yields by promoting dry matter production, productive spike number and 1000-grain weight (**Tables 3**, **4**). Higher grain yield was determined by the higher count of productive spikes, grains per spike, and grain weight (Chen et al., 2023). The number of grains per spike and the weight of each grain are associated with dry matter production (Chen et al., 2023; Wang et al., 2021). One reason for increasing the yield is the use of CRU, which has a lower N release rate during early crop growth, when N requirements are relatively low and provides a sustained nutrient supply throughout the entire crop period as compared to urea (Cui et al., 2022). Another reason is the use of double-layer fertilization, plants get more nutrients from the upper soil layer (8–10 cm) when they are in their initial growth stages, but in later stages, they rely on roots to get nutrients from the deeper soil layer (Ma et al., 2021b; Wang et al., 2023). Our results also indicated that N fertilizer types and layer fertilization are positively correlated with grain yield (**Figure 10**). Moreover, grain yield, spike number, grains per spike, 1000-grain weight, and dry matter production show strong positive interrelationships (**Figure 9**). These findings suggest that one-time double-layer fertilization of CRU (T4) significantly contributes to enhancing grain yield in winter wheat.

The emission of N_2_O from agricultural soil is mostly affected by N fertilizer type (control release and normal urea), amount (≥180 kg ha^-1^) and method (shallow surface and deep) (Aliyu et al., 2019; Ding et al., 2007; Ma et al., 2021b). In this study, the types (normal urea vs. CRU) and techniques of N-layer fertilization (double-layer vs. single-layer) primarily influenced N_2_O emissions. Previous research indicated that the single-layer application of N had varying impacts on N_2_O emissions (Chen et al., 2023; Gaihre et al., 2015; Ke et al., 2018; Ma et al., 2021b; Wang et al., 2023; Wu et al., 2021; Zhang et al., 2022). Shallow single-layer application of N fertilizer increases N_2_O emissions, whereas deeper single-layer application reduces N_2_O emissions but poses a higher risk of N leaching (Ke et al., 2018; Liu et al., 2024a; Wang et al., 2023). The main reason for the findings of previous studies could be the use of traditional urea, which tends to accumulate in the root zone. A higher concentration of NH_4_^+^-N can be changed into NO_3_^−^-N due to high soil temperature and low moisture, which is a substrate for nitrification and releases N_2_O (Song et al., 2018; Wu et al., 2021; Zhang et al., 2022). In our study, we observed that one-time double-layer fertilization of CRU (T4) reduced the total N_2_O, yield scale N_2_O and emission factor (**Figures 5**, **6**) by decreasing the concentration of N in the 0–10 cm soil layer and the anaerobic condition caused by optimum soil moisture may restrict the conversion of NH_4_^+^-N into NO_3_^−^-N (**Figures 2**, **3**, **7**). The mitigation of N_2_O emissions is attributed to the application of a double-layer, which optimizes soil moisture and reduces the temperature at deeper soil layers. As a result, the conversion of NH_4_^+^-N into NO_3_^−^-N is decreased (Daly et al., 2024; Liu et al., 2024b; Toma et al., 2007; Wang et al., 2023). In addition, the N need of wheat was minimal throughout the early growth period, but there was a substantial demand for N from the jointing to the heading stage (Tian et al., 2018). Hence, the use of CRU synchronizes fertilizer application with the specific nutrient requirements of the crops compared to urea and minimizes the loss of N_2_O. Analysis of NO_3_^−^-N levels during various stages of wheat growth revealed that double-layered fertilization, as opposed to single-deep fertilization, led to the formation of continuous high-concentration NH_4_^+^-N in the 10–20 cm soil layer (**Figures 7**, **8**). The inverse relationship between gaseous N_2_O emissions and the types of layer fertilization in agricultural environments causes this phenomenon (**Figure 10**). N_2_O emission and soil NO_3_^−^-N concentration at various soil depths showed a positive correlation, suggesting that the low N_2_O emissions of the double-layer CRU are mostly caused by the low NO_3_^−^-N concentration in the top (0–10 cm) soil layer (**Figure 9**).

Nitrogen use efficiency (NUE) is a fundamental metric for assessing the N uptake, recovery and utilization efficiency of crops (Chen et al., 2023). Previous studies suggest that the optimal strategy for enhancing NUE is to apply N fertilizer specifically to the soil layer that is 10–15 cm deep (Wu et al., 2021). Some other researches show that application of N fertilizers at the upper soil layer has a negative impact on NUE due to an initial boost the crop productivity followed by a subsequent reduction (Chen et al., 2023; Wang et al., 2023). However, applying too much N fertilizer in deeper soil layers may overestimate a crop N requirement, potentially restricting N availability for seedlings (Ma et al., 2021a). Soil nutrient availability increases with rising moisture levels, leading to enhanced plant nutrient absorption. However, once a certain moisture level is attained, nutrient availability begins to decline (Zhang et al., 2020). The strategic positioning of layer fertilizer application at a soil depth resulted in a prolonged supply of N and increased NUE, but there is a research gap between the one-time layer fertilization (single-layer vs. double-layer) of different N fertilizers (urea vs. CRU). In our study, we observed that one-time double-layer fertilization of CRU (T4) increased the NUE by enhancing the NRE, PFPN and N uptake (**Tables 2**, **5**). The CRU reduces N release rate during early crop growth, when N requirements are relatively low and provides a sustained nutrient supply throughout the entire crop period, thereby significantly enhancing the NUE (Cui et al., 2022). The CRU enhanced the N uptake by reducing the conversion of NH_4_^+^-N into NO_3_^−^-N (Geng et al., 2016). Our results indicated that N fertilizer types and layered fertilization are positively correlated with N uptake (**Figure 10**). Moreover, The NUE and the plant N uptake are positively correlated with each other but negatively correlated with NO_3_^−^-N (**Figure 9**). Additionally, the transformation of soil N undergoes biological activities that are influenced by soil temperature and moisture (Song et al., 2018). In our research, we observed significant differences in the hydrothermal conditions between the double-layer fertilization of CRU, due to optimum moisture and low temperature (**Figures 2**, **3**). In this scenario, both the release and the length of the soil availability of N can be prolonged with the double-layer placement of CRU. This was also confirmed by previous research that soil moisture significantly impacts the NUE and N absorption (Liu et al., 2022b, 2023, 2017).

**Figure 11:**
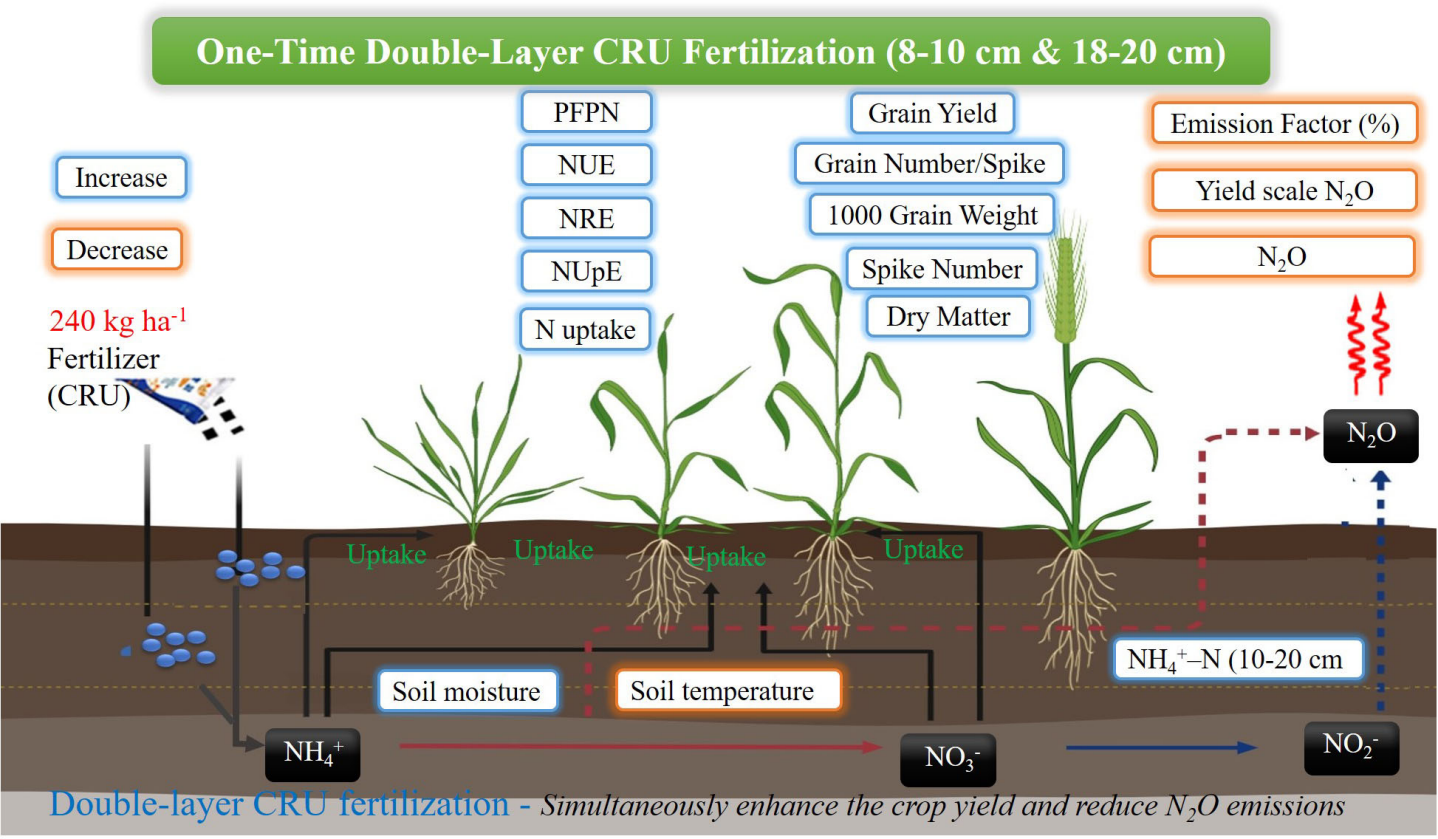
A conceptual framework for elucidating the mechanism of layered N fertilizer application.

The improvement in living standards in China has raised food consumption and increased the demand for higher-quality products in recent decades (Han et al., 2023). Nevertheless, a shortage of resources including nutrients, arable land, energy, and water, poses a subnational challenge to the potential increase in crop production (Hu et al., 2023b). To address these challenges, Chinese agricultural researchers and policymakers aim to improve fertilization strategies to boost crop yields and minimize environmental pollution. Farmers are adopting innovative fertilizing methods to reduce production costs (Hu et al., 2023b; Wu et al., 2021; Zhang et al., 2022). Among these, modifying N fertilizer types and layered placement during sowing has emerged as a practical and effective approach. A single application of CRU fertilizer and single-layer placement at 8–10 cm soil depth makes this strategy promising in China. However, our one-time double-layer fertilization strategy must be suitable for the fertilization equipment used for agricultural output. Some areas have successfully built deep fertilizer application machinery in recent years (Cui et al., 2022; Geng et al., 2016; Xu et al., 2021), which could help spread the use of this approach. A framework is proposed to explain the mechanism behind layered N fertilization, designed to improve the coordination of both yield and NUE, while reducing N_2_O emissions in winter wheat, as illustrated in **Figure 11**. Our findings indicated that the application of one-time double-layer fertilization of CRU have proven to be more successful than either single-layer or double-layer urea fertilizer treatment. However, before the adoption of this approach by farmers, it is essential to evaluate its potential acceptance and associated costs across various environmental conditions and crop systems. Farmers in underdeveloped nations may be less aware of the environmental implications of fertilization due to lower educational levels; instead, they may concentrate on the higher fuel costs associated with double-layer fertilizer application. Therefore, governments must adopt suitable strategies to motivate farmers to actively utilize this technology, while also facilitating their comprehension of the equal significance of economic revenue and environmental conservation and offering adequate subsidies to enhance agricultural productivity and income levels.

## 5 Conclusion

The layered fertilization of different N fertilizer significantly affects the crop growth, yield, and N use efficiency by regulating the soil inorganic N content and mitigating N_2_O emissions. The types of N fertilizers and its layer-specific fertilization impact the dry matter production, crop N uptake, yield components and NUE-related parameters, collectively promoting the yield of winter wheat. Among these strategies, utilizing the one-time double-layer fertilization of CRU at 8–10 cm & 18–20 cm soil depth has proven to be the most effective for maximum winter wheat production and minimizing environmental impacts. This approach synchronizes N release with crop demand, optimizing N uptake and reducing N_2_O emissions. Given its potential to improve both crop yield and environmental sustainability, the adoption of double-layer CRU fertilization should be encouraged through targeted policy interventions and farmer incentives. Further research is needed to assess the long-term effects of this method across different soil types, crops, and environmental conditions, and to evaluate its economic feasibility for broader implementation in diverse agricultural settings.

## Data Availability

The original contributions presented in the study are included in the article/supplementary material. Further inquiries can be directed to the corresponding authors.
